# New Wing of South London Hospital

**Published:** 1924-07

**Authors:** 


### NEW WING OF SOUTH LONDON HOSPITAL.

The Duchess of York recently opened the new wing of the South London Hospital for Women on Clapham Common. Sir Henry Jackson, M.D., in a speech of welcome, spoke of the admirable work of the hospital and of its doctors and nurses—" a splendid band of women who day and night are fighting disease in this hospital, which is indeed a ' house of hospitality.' " Next, fifty-five purses of money, the value of which totalled £1,100, were handed to Her Royal Highness by pretty white-clad children. Mr. E. L. Franklin made a speech of thanks and gave a brief financial statement. Her Royal Highness then drew a cord which opened the doors of the new wing and declared it open. It was dedicated by the Bishop of Kingston.

### From Club to Hospital.

The new wing, which was originally a Conservative club known as Preston House, was added through the generosity of anonymous friends, who bought the freehold and vested it in the Hospital Trustees, in addition to which they contributed £12,000 towards the cost of the necessary alterations. It is set well back from the road, having a north-westerly aspect with a fine view of Clapham Common. There is a good garden at the back which affords a recreation ground and a tennis court for the nurses. Inside it is all that is modern, hygienic, airy and pleasing to the eye, from the third and top floor with its anæsthetising room adjoining a well-equipped theatre and its night-nurses' bedrooms to the ground floor with its bright white and blue ward and its cubicles, where private patients will be accommodated at about five guineas a week. All floors are reached by means of a lift and a broad fire-resisting staircase, while fire-resisting doors and an iron external staircase practically eliminate any danger from fire. By means of the thirty-six additional beds afforded by the new wing it is hoped considerably to reduce the hospital's waiting list of over 600 names.

